# Body image and quality of life in patients with and without body contouring surgery following bariatric surgery: a comparison of pre- and post-surgery groups

**DOI:** 10.3389/fpsyg.2014.01310

**Published:** 2014-11-18

**Authors:** Martina de Zwaan, Ekaterini Georgiadou, Christine E. Stroh, Martin Teufel, Hinrich Köhler, Maxi Tengler, Astrid Müller

**Affiliations:** ^1^Department of Psychosomatic Medicine and Psychotherapy, Hannover Medical SchoolHannover, Germany; ^2^Department of General, Abdominal and Pediatric Surgery, SRH Wald-Klinikum GeraGera, Germany; ^3^Department of Psychosomatic Medicine and Psychotherapy, Medical University Hospital, University of TuebingenTuebingen, Germany; ^4^Department of Surgery, Herzogin Elisabeth HospitalBraunschweig, Germany

**Keywords:** body image quality of life, depression, anxiety, body contouring surgery, bariatric surgery

## Abstract

**Background:** Massive weight loss (MWL) following bariatric surgery frequently results in an excess of overstretched skin causing physical discomfort and negatively affecting quality of life, self-esteem, body image, and physical functioning.

**Methods:** In this cross-sectional study 3 groups were compared: (1) patients prior to bariatric surgery (*n* = 79), (2) patients after bariatric surgery who had not undergone body contouring surgery (BCS) (*n* = 252), and (3) patients after bariatric surgery who underwent subsequent BCS (*n* = 62). All participants completed self-report questionnaires assessing body image (Multidimensional Body-Self Relations Questionnaire, MBSRQ), quality of life (IWQOL-Lite), symptoms of depression (PHQ-9), and anxiety (GAD-7).

**Results:** Overall, 62 patients (19.2%) reported having undergone a total of 90 BCS procedures. The most common were abdominoplasties (88.7%), thigh lifts (24.2%), and breast lifts (16.1%). Post-bariatric surgery patients differed significantly in most variables from pre-bariatric surgery patients. Although there were fewer differences between patients with and without BCS, patients after BCS reported better appearance evaluation (AE), body area satisfaction (BAS), and physical functioning, even after controlling for excess weight loss and time since surgery. No differences were found for symptoms of depression and anxiety, and most other quality of life and body image domains.

**Discussion:** Our results support the results of longitudinal studies demonstrating significant improvements in different aspects of body image, quality of life, and general psychopathology after bariatric surgery. Also, we found better AE and physical functioning in patients after BCS following bariatric surgery compared to patients with MWL after bariatric surgery who did not undergo BCS. Overall, there appears to be an effect of BCS on certain aspects of body image and quality of life but not on psychological aspects on the whole.

## Introduction

In Germany about 1.5% of the population is extremely obese (de Zwaan, 2012). In 2012, 6678 primary bariatric surgeries were reported to the German Bariatric Surgery Registry which contains information on approximately three fourth of all bariatric surgeries performed in Germany (Stroh et al., 2014). The most frequently employed procedures were laparoscopic gastric bypass and sleeve gastrectomy. As in other countries the number of surgeries has been rapidly increasing over the last years.

Bariatric surgery represents the only effective treatment for extreme obesity and leads to significant and long-term weight reduction with a concomitant significant improvement in overall quality of life (Buchwald et al., 2004; Karlsson et al., 2007). However, massive weight loss (MWL) following bariatric surgery frequently results in redundant skin folds which can lead to difficulty in mobilizing and exercising and may lead to intertrigo, ulceration, and infection. Excess skin is predominantly found on the upper arms, breasts, abdomen, and thighs. The resulting deformities cannot be addressed adequately with exercise or diets (Colwell, 2010). Loose and hanging skin following MWL has shown to negatively impact quality of life, self-esteem, body image, and physical functioning (Kinzl et al., 2003; Sarwer et al., 2008; Klassen et al., 2012). Klassen et al. (2012) conducted in-depth interviews with 43 individuals who underwent body contouring surgery (BCS) following weight loss. Participants described feeling socially isolated, being limited in performing their usual social roles, and even feeling “like a freak” due to deformities of body regions. However, only a minority of patients (4–5%) would not undergo bariatric surgery again due to development of excess skin (Kitzinger et al., 2012).

Thus, paralleling the increase in bariatric surgery, there is also a greater desire for subsequent BCS (Mitchell et al., 2008; Aldaqal et al., 2012; Kitzinger et al., 2012; Steffen et al., 2012; Mukherjee et al., 2014). The most common procedures are abdominoplasty, breast lift, upper arm lift, thigh lift, and lower body lift. A number of studies have shown that up to 80% of post-bariatric surgery patients desire BCS, often in several areas, with women usually having a stronger desire (Kitzinger et al., 2012; Aldaqal et al., 2013). Only about 20% actually undergo these procedures (Giordano et al., 2014). Thus, there is a marked discrepancy between the number of subjects who indicate that they desire such surgery and those who actually receive it. This may be a function of financial resources and coverage from third-party payers. However, also in countries where the costs are usually covered, a large gap exists (Kitzinger et al., 2012). This might be due to fear of complications of sequential operations and scarring. Indeed, a recent meta-analysis demonstrated that there is a 60–80% increased risk of developing complications after BCS when the weight was lost after bariatric surgery compared to weight loss due to dietary changes (Hasanbegovic and Sørensen, 2014). However, there is evidence that the occurrence of post-operative complications does not significantly influence patient satisfaction with the final result of BCS (van der Beek et al., 2010).

Even though one might assume that reconstructive surgery will trigger changes in body image and quality of life that exceed the improvements due to MWL after bariatric surgery, the results in the literature are somewhat mixed. Indeed, most studies found improvements after BCS in quality of life, body image, self-esteem, and sexuality (Song et al., 2006; Pecori et al., 2007; van der Beek et al., 2010, 2012; Bracaglia et al., 2011; Modarressi et al., 2013). There is also evidence that BCS improves weight control after bariatric surgery (Balagué et al., 2013). In addition, BCS not only improves the aesthetic outcome of bariatric surgery it also corrects functional impairment (van der Beek et al., 2010; Coriddi et al., 2011). However, some, albeit fewer, studies did not find significant differences in quality of life measures between post-bariatric surgery patients with and without subsequent BCS (Song et al., 2006; Singh et al., 2012).

There is still more research needed on psychosocial aspects of BCS after bariatric operations. The goal of our study was to investigate if BCS has an impact on psychosocial variables that exceeds the impact that bariatric surgery exerts on those variables. We approached this question by comparing 3 large cohorts of patients on a variety of measures on body image, quality of life, symptoms of depression and anxiety, using a cross-sectional design. Three groups were compared: (1) patients prior to bariatric surgery (*n* = 79), (2) patients at least 1 year after bariatric surgery who had not undergone BCS (*n* = 252), and (3) patients at least 1 year after bariatric surgery who also underwent subsequent BCS (*n* = 62). We expected significant differences between pre- and post-bariatric surgery patients on all psychological variables. In addition we hypothesized that patients after BCS would evaluate their appearance more positively and would report better physical functioning compared to patients after bariatric surgery who did not undergo BCS. However, with regard to the other psychosocial variables we did not expect significant differences between patients with and without BCS.

## Materials and methods

### Participants

Patients before and after bariatric surgery were asked to complete several self-report questionnaires. Data were collected from a total of 393 participants. Of those, 79 consecutive patients prior to bariatric surgery received the questionnaires before they were scheduled for their psychiatric evaluation at the Department of Psychosomatic Medicine and Psychotherapy at Hannover Medical School. The clinicians performing the psychiatric evaluation were not informed about the results of the assessment. 314 patients completed the survey at least 1 year after bariatric surgery (1–15.5 years). The patients after bariatric surgery were recruited at three different sites (Department of Surgery, Herzogin Elisabeth Hospital, Braunschweig; Department of Surgery, SRH Wald-Klinikum Gera, and Department of Psychosomatic Medicine, University Hospital Tuebingen). They were either given the questionnaires during one of their routine follow-up visits at the respective Surgery Department or were sent the questionnaires by mail. A cover letter was included explaining the study as well as a consent form. The study was approved by the Institutional Review Boards of Hannover Medical School and all patients gave written informed consent.

### Measures

#### Sociodemographics

Age, sex, socio-demographic variables (marital status, educational status), weights and height pre- and post-surgery, and duration (in months) since bariatric surgery were self-reported. Pre-bariatric surgery body mass index (BMI), current BMI, minimal BMI since bariatric surgery, percent weight loss (%WL), and percent excess weight loss (%EWL) relative to a BMI of 25 kg/m^2^ were calculated.

#### Body image

Body image was investigated using the Multidimensional Body-Self Relations Questionnaire (MBSRQ; Cash et al., 1991; Brown et al., 1999), a validated, 69 item self-report inventory which consists of 10 subscales assessing multiple aspects of body image. The MBSRQ has been used before in individuals prior to and after weight loss (Foster et al., 1997) and prior to and after abdominoplasty (Bolton et al., 2003; Singh and Losken, 2011) and, thus, was considered a feasible instrument for or study.

The MBSRQ is able to differentiate between the “evaluation” of appearance-related aspects and the person's “orientation” toward these aspects (i.e., the perceived importance of appearance and its influence on the person's behavior). The subscales are Appearance Evaluation (AE, Cronbach's α in our sample 0.85), Fitness Evaluation (FE, Cronbach's α 0.77), Health Evaluation (HE, Cronbach's α 0.83), Appearance Orientation (AO, Cronbach's α 0.83), Fitness Orientation (FO, Cronbach's α 0.81), Health Orientation (HO, Cronbach's α 0.64), and Illness Orientation (IO). In addition, the MBSRQ has three special subscales: (1) The Body Areas Satisfaction scale (BAS) assessing the satisfaction with different body areas and attributes (Cronbach's α in our sample 0.75), (2) the Overweight Preoccupation scale (OP) assessing fat anxiety, weight vigilance, dieting, and eating restraint (Cronbach's α 0.40), and (3) the Self-Classified Weight scale (SCW) assessing self-appraisals of weight (Cronbach's α 0.89). Most items measure agreement (1 = definitely disagree to 5 = definitely agree), satisfaction (1 = very dissatisfied to 5 = very satisfied), or frequency (1 = never to 5 = very often). The SCW scale has five specific response options (1 = very underweight to 5 = very overweight). US adult norms for the questionnaire are available from *n* = 996 males and *n* = 1070 females. In the female norm sample, Cronbach's alpha ranged from 0.73 to 0.89 for the different subscales (Cash, 2000).

A German version of the 34 item Appearance Subscale version of the questionnaire (MBSRQ-AS; Cash, 2000) which contains the scales AE (7 items), AO (12 items), BAS (9 items), OP (4 items), and SCW (2 items) is available and was validated in eating disordered patients (Vossbeck-Elsebusch et al., 2014). The remaining subscales were also translated and back-translated by the same group, and have shown good to acceptable internal consistency in our sample.

#### Satisfaction with body regions

In addition, all post-bariatric surgery patients were asked to rate their current satisfaction with different body regions on a 7-point Likert scale (from “very satisfied” to “very dissatisfied”). Patients who underwent BCS were also asked to rate their current satisfaction with the contoured body regions. In addition, they were asked to retrospectively rate their satisfaction with the contoured regions prior to the operation on the same 7 point Likert scale ranging from “very dissatisfied” to “very satisfied.”

#### Quality of life

The Impact of Weight on Quality of Life Questionnaire (IWQOL-Lite; Kolotkin et al., 2001) is a weight-specific measure of HRQOL. The instrument consists of 31 items which focus on concerns of overweight/obese individuals. Individuals are asked to answer each item on a 5-point scale ranging from “never true” to “always true.” A total score and 5 subscale scores can be calculated: Physical Function, Self-Esteem, Sexual Life, Public Distress, and Work. Internal reliabilities (α) for the subscales in our sample were 0.96, 0.96, 0.92, 0.94, and 0.87. A validated German version is available (Mueller et al., 2011). Lower scores indicate less impairment and better quality of life.

#### Symptoms of depression and anxiety

Symptoms of depression were assessed with the German version of the 9-item Patient Health Questionnaire-Depression Scale (PHQ-9; Spitzer et al., 1999; Löwe et al., 2004). Each item is scored from 0 to 3, yielding a total score between 0 and 27. A total score ≥10 indicates the presence of a major depressive disorder (MDD). The PHQ has been validated in bariatric surgery patient populations (Cassin et al., 2013) and has been used before to compare bariatric surgery patients with and without BCS (Azin et al., 2014). Cronbach's α in the present study sample was 0.87.

Symptoms of anxiety were assessed with the German version of the 7-item Generalized Anxiety Scale (GAD-7, Spitzer et al., 2006; Löwe et al., 2008). The items are also scored from 0 to 3, yielding a total score between 0 and 21. The GAD-7 has been used before to compare bariatric patients with and without BC surgery (Azin et al., 2014). Cronbach's α in the present study sample was 0.90.

The scores of the questionnaires in the post-bariatric surgery groups did not differ between sites (data not shown).

### Statistics

All statistical analyses were performed using SPSS for Windows version 21.0 (SPSS, Inc., Chicago, Ill.).

The results of the questionnaires were compared between the three groups (pre-bariatric surgery, post-bariatric surgery without BC surgery, and post-bariatric surgery after BC surgery) using One-Way analyses of variance (ANOVAs) and Tukey-B *post-hoc* tests for continuous variables and chi-square tests for ordinal or dichotomous variables. Adjustments were performed using age and symptoms of depression as controlling variables. The differences between groups were expressed by way of partial eta-squared.

In addition, the post-bariatric surgery groups with and without BC surgery were compared using *t*-tests and ANOVAs controlling for time since surgery and %EWL.

Since not all variables met the assumption of normality the comparison between groups were repeated using non-parametric tests (Wilcoxon and Mann–Whitney *U*-tests).

A value of *p* < 0.05 was considered statistically significant.

## Results

### Comparison between pre- and post-bariatric surgery patients

Significant differences were found between pre- and post-bariatric surgery patients in almost all variables using parametric tests (Tables 1, 2). Non-parametric tests confirmed the results. Compared to pre-bariatric surgery patients the post-bariatric surgery patients exhibited a lower BMI, were older, reported less symptoms of depression and anxiety, a better quality of life in all domains, and improved body image in all but 2 (AO, IO) subscales of the MBSRQ. After controlling for depressive symptoms only the significant differences in OP disappeared; controlling for age did not change any of the results.

**Table 1:** **Quality of life, symptoms of depression and anxiety: comparison between pre- and post-bariatric surgery groups with and without subsequent BCS**.

	**Pre-bariatric surgery**	**Post-surgery no body contouring**	**Post-surgery and body contouring**	**Statistics**	**Adjusted for age**	**Adjusted for depressive symptoms**
	***M* (*SD*)**	***M* (*SD*)**	***M* (*SD*)**			
BMI current (kg/m^2^)	48.74^a^ (7.97)	34.46^b^ (7.31)	32.46^b^ (6.07)	*F*_(2, 389)_ = 130.53 *p* < 0.001; η^2^ = 0.40	*F*_(2, 387)_ = 136.61 *p* < 0.001; η^2^ = 0.41	*F*_(2, 385)_ = 105.54 *p* < 0.001; η^2^ = 0.35
Age	41.63^a^ (10.40)	47.91^b^ (11.30)	47.97^b^ (9.93)	*F*_(2, 389)_ = 10.74 *p* < 0.001; η^2^ = 0.05	–	*F*_(2, 385)_ = 7.99 *p* < 0.001; η^2^ = 0.04
Symptoms of anxiety (GAD-7 total score)	8.00^a^ (4.89)	5.93^b^ (5.21)	5.41^b^ (4.74)	*F*_(2, 389)_ = 6.02 *p* = 0.003; η^2^ = 0.03	*F*_(2, 387)_ = 4.46 *p* = 0.012; η^2^ = 0.02	*F*_(2, 386)_ = 9.51 *p* < 0.001; η^2^ = 0.05
Symptoms of depression (PHQ-9 total score)	12.43^a^ (5.32)	7.24^b^ (5.80)	7.09^b^ (5.17)	*F*_(2, 387)_ = 26.99 *p* < 0.001; η^2^ = 0.12	*F*_(2, 385)_ = 24.31 *p* < 0.001; η^2^ = 0.11	–
IWQOL total^*^	118.91^a^ (23.03)	64.50^b^ (27.95)	58.94^b^ (25.81)	*F*_(2, 390)_ = 137.43 *p* < 0.001; η^2^ = 0.41	*F*_(2, 388)_ = 134.98 *p* < 0.001; η^2^ = 0.41	*F*_(2, 386)_ = 104.38 *p* < 0.001; η^2^ = 0.35
IWQOL physical function^*^	45.13^a^ (8.50)	22.90^b^ (11.22)	19.36^c^ (9.15)	*F*_(2, 389)_ = 155.26 *p* < 0.001; η^2^ = 0.44	*F*_(2, 387)_ = 184.10 *p* < 0.001; η^2^ = 0.49	*F*_(2, 385)_ = 112.12 *p* < 0.001; η^2^ = 0.37
IWQOL self-esteem^*^	28.89^a^ (6.27)	17.20^b^ (8.50)	16.26^b^ (8.88)	*F*_(2, 389)_ = 66.92 *p* < 0.001; η^2^ = 0.26	*F*_(2, 387)_ = 57.37 *p* < 0.001; η^2^ = 0.23	*F*_(2, 385)_ = 38.26 *p* < 0.001; η^2^ = 0.17
IWQOL sexual life^*^	13.42^a^ (5.07)	8.94^b^ (5.27)	8.58^b^ (5.20)	*F*_(2, 381)_ = 22.77 *p* < 0.001; η^2^ = 0.10	*F*_(2, 379)_ = 21.71 *p* < 0.001; η^2^ = 0.10	*F*_(2, 378)_ = 7.83 *p* < 0.001; η^2^ = 0.04
IWQOL public distress^*^	17.41^a^ (5.50)	8.64^b^ (4.90)	8.03^b^ (4.43)	*F*_(2, 388)_ = 101.89 *p* < 0.001; η^2^ = 0.34	*F*_(2, 386)_ = 90.25 *p* < 0.001; η^2^ = 0.32	*F*_(2, 385)_ = 69.06 *p* < 0.001; η^2^ = 0.26
IWQOL work^*^	12.86^a^ (4.58)	6.47^b^ (3.52)	5.76^b^ (2.76)	*F*_(2, 386)_ = 100.70 *p* < 0.001; η^2^ = 0.34	*F*_(2, 384)_ = 98.41 *p* < 0.001; η^2^ = 0.34	*F*_(2, 383)_ = 65.24 *p* < 0.001; η^2^ = 0.25

*Values with different superscripts are significantly different (Tukey-B post-hoc tests). η^2^ = eta-squared (effect size)*.

* *Lower scores indicate less impairment = better quality of life*.

*Abbreviation: IWQOL, Impact of Weight on Quality of Life-Lite*.

**Table 2:** **Body image: comparison between pre- and post-bariatric surgery groups with and without subsequent BCS**.

	**Pre-bariatric surgery**	**Post-surgery no body contouring**	**Post-surgery and body contouring**	**Statistics**	**Adjusted for age**	**Adjusted for depression**
	***M* (*SD*)**	***M* (*SD*)**	***M* (*SD*)**			
MBSRQ_AE	1.46^a^ (0.47)	2.47^b^ (0.80)	2.73^c^ (0.84)	*F*_(2, 380)_ = 63.76 *p* < 0.001; η^2^ = 0.25	*F*_(2, 378)_ = 53.62 *p* < 0.001; η^2^ = 0.22	*F*_(2, 378)_ = 36.50 *p* < 0.001; η^2^ = 0.16
MBSRQ_AO	3.50^a^ (0.71)	3.68^a^ (0.69)	3.66^a^ (0.65)	*F*_(2, 381)_ = 2.01 *p* = 0.135; η^2^ = 0.01	*F*_(2, 379)_ = 4.55 *p* = 0.011; η^2^ = 0.02	*F*_(2, 378)_ = 3.74 *p* = 0.025; η^2^ = 0.02
MBSRQ_FE	2.72^a^ (0.82)	3.26^b^ (0.85)	3.38^b^ (0.83)	*F*_(2, 373)_ = 13.60 *p* < 0.001; η^2^ = 0.07	*F*_(2, 371)_ = 15.13 *p* < 0.001; η^2^ = 0.07	*F*_(2, 370)_ = 6.32 *p* = 0.002; η^2^ = 0.03
MBSRQ_FO	2.91^a^ (0.63)	3.26^b^ (0.67)	3.26^b^ (0.71)	*F*_(2, 376)_ = 8.27 *p* < 0.001; η^2^ = 0.04	*F*_(2, 374)_ = 10.26 *p* < 0.001; η^2^ = 0.05	*F*_(2, 373)_ = 3.79 *p* = 0.023; η^2^ = 0.02
MBSRQ_HE	2.19^a^ (0.71)	3.06^b^ (0.93)	3.09^b^ (0.89)	*F*_(2, 382)_ = 30.50 *p* < 0.001; η^2^ = 0.14	*F*_(2, 380)_ = 31.04 *p* < 0.001; η^2^ = 0.14	*F*_(2, 379)_ = 9.13 *p* < 0.001; η^2^ = 0.05
MBSRQ_HO	3.16^a^ (0.57)	3.53^b^ (0.55)	3.53^b^ (0.57)	*F*_(2, 383)_ = 13.80 *p* < 0.001; η^2^ = 0.07	*F*_(2, 381)_ = 10.75 *p* < 0.001; η^2^ = 0.05	*F*_(2, 380)_ = 7.86 *p* < 0.001; η^2^ = 0.04
MBSRQ_IO	2.82^a^ (0.72)	2.99^a^ (0.69)	2.83^a^ (0.56)	*F*_(2, 381)_ = 2.28 *p* = 0.103; η^2^ = 0.01	*F*_(2, 379)_ = 2.26 *p* = 0.105; η^2^ = 0.01	*F*_(2, 378)_ = 1.99 *p* = 0.138; η^2^ = 0.01
MBSRQ_BAS	2.15^a^ (0.50)	2.81^b^ (0.62)	3.04^c^ (0.62)	*F*_(2, 387)_ = 47.55 *p* < 0.001; η^2^ = 0.20	*F*_(2, 385)_ = 45.27 *p* < 0.001; η^2^ = 0.19	*F*_(2, 383)_ = 24.94 *p* < 0.001; η^2^ = 0.11
MBSRQ_OP^**^	3.61^a^ (0.74)	3.22^b^ (0.78)	3.33^b^ (0.82)	*F*_(2, 371)_ = 6.64 *p* = 0.001; η^2^ = 0.03	*F*_(2, 369)_ = 6.98 *p* < 0.001; η^2^ = 0.04	*F*_(2, 367)_ = 2.31 *p* = 0.100; η^2^ = 0.01
MBSRQ_SCW^**^	4.91^a^ (0.48)	4.20^b^ (0.70)	3.92^c^ (0.80)	*F*_(2, 382)_ = 43.90 *p* < 0.001; η^2^ = 0.19	*F*_(2, 380)_ = 48.37 *p* < 0.001; η^2^ = 0.20	*F*_(2, 379)_ = 27.22 *p* < 0.001; η^2^ = 0.12

*Values with different superscripts are significantly different (Tukey-B post-hoc tests). η^2^ = eta-squared (effect size)*.

** *Higher scores indicate more preoccupation with overweight (OP) and a more obese self-perception (SCW); for all other MBSRQ subscales higher scores indicate a more positive body image*.

*Abbreviations: MBSRQ, Multidimensional Body-Self Relations Questionnaire; AE, Appearance Evaluation; AO, Appearance Orientation; FE, Fitness Evaluation; FO, Fitness Orientation; HE, Health Evaluation; HO, Health Orientation; IO, Illness Orientation; BAS, Body Areas Satisfaction; OP, Overweight Preoccupation; SCW, Self-Classified Weight*.

With regard to depression the percentages of patients above the proposed cutoff of 10 on the PHQ-9 were 71.8% in the pre-bariatric surgery group (*n* = 56), 30.8% in the post-bariatric surgery group who did not undergo BCS (*n* = 77), and 27.4% in the body contouring group (*n* = 17) (χ^2^ = 46,011, *df* = 2, *p* < 0.001).

### Description of participants who had undergone BCS

Of the 314 post-bariatric surgery patients 175 (55.9%) underwent laparoscopic gastric bypass, 71 (22.7%) sleeve gastrectomy, 63 (20.1%) adjustable gastric banding, and 4 (1.3%) another type of surgery.

Overall, 62 (19.7%) underwent BCS. The vast majority of post-bariatric surgery patients who underwent BCS had an abdominoplasty (55; 88.7%). Other common BC procedures were thigh lifts (15, 24.2%) and breast lifts (10, 16.1%). Six patients (9.7%) had surgery on their arms, 1 patient on the upper back, 7 (11.3%) patients on the lower back, and 4 (6.5%) patients on their buttocks. Overall, BCS patients reported a mean of 1.45 (1–5) plastic surgery procedures on 1.6 (1–5) body regions. 42 patients underwent a single reconstructive procedure, 8 underwent two operations, 8 of the patients underwent 3 operations, 3 underwent 4, and 1 patient underwent 5 operations. Abdominoplasty was covered by third party payers in 49 patients (89.1%) as were 80% of the thigh lifts but only 40% of the breast lifts.

Of the 55 patients who underwent an abdominoplasty 51 (92.7%) reported to have been very dissatisfied or dissatisfied with their abdomen prior to the operation. After the contouring surgery 17 (30.9%) reported to be very satisfied or satisfied with the result; however, 15 (27.2%) were still very dissatisfied or dissatisfied. All patients with thigh lifts reported to have been (very) dissatisfied with this body region prior to BCS; however, 6 patients (40%) were still very dissatisfied or dissatisfied after the operation. None of the patients with breast lifts were dissatisfied with the BCS result whereas 90% reported to have been very dissatisfied prior to the operation.

### Comparison between post-bariatric surgery patients with and without subsequent BCS

We did not find differences in educational level, marital status, and gender distribution between bariatric surgery patients with and without BCS. Of the post-bariatric surgery patients who underwent subsequent BCS 85.2% were female, 55.7% were married, and 14.8% had finished high school. The respective percentages in patient without BCS were 78.2, 58.8, and 9.8%. Also, the groups did not differ with regard to current BMI and minimal BMI since bariatric surgery. However, groups differed significantly with regard to time (months) since surgery (*t* = −3.091, *df* = 307, *p* = 0.002), %WL (*t* = −3.051. *df* = 310. *P* = 0.002), and %EWL (*t* = −3.120, *df* = 310, *p* = 0.002). Patients without BCS (*n* = 252) were assessed 37.8 months (12–185) after bariatric surgery and reported to have lost 31.7% of their weight and 65.5% of their excess weight. Patients who underwent BCS (*n* = 62) were assessed 49.8 months (14–188) after bariatric surgery and had lost 36.9% of their weight and 79.1% of their excess weight.

As shown in Tables 1, 2, BCS patients reported significantly better Physical Functioning, AE, BAS, and a lower SCW even though the current BMIs were not different between groups. Separate analyses of the nine items of the BAS scale revealed that the mid-torso (abdominal) area was actually the only region significantly differing between groups. No differences were found for symptoms of depression and anxiety, 4 of the 5 IWQOL-Lite subscales, and 7 of the 10 MBSRQ subscales.

Since most post-bariatric surgery patients who underwent abdominoplasty, breast, or thigh lifts reported that they were (very) dissatisfied with the respective body region prior to BCS surgery (90–100%), we compared patients who received BCS with those bariatric surgery patients who did not receive BCS but reported to be very dissatisfied or dissatisfied with the respective body region. Of the 252 post-bariatric surgery patients who had not undergone BCS 183 (72.6%) reported to be very dissatisfied or dissatisfied with their abdominal region, 141 (55.9%) were very dissatisfied or dissatisfied with their thighs, and 134 (53.1%) with their breasts. Since the number of patients who received breast lifts (*n* = 10) and thigh lifts (*n* = 15) were rather small, we only compared patients after abdominoplasty (*n* = 54) with patients who had not received abdominoplasty following bariatric surgery but who were (very) dissatisfied with their abdominal region (*n* = 183) (Table 3). Age, BMI, and sex (females: 82.5 vs. 83%) distribution did not differ between groups. We found more differences between these two specific groups than in the analyses including all post-bariatric patients. In addition to Physical Functioning, most other quality of life domains also showed significant differences, e.g., patients after abdominoplasty reported significantly better self-esteem, a more satisfying sexual life, and a better work performance compared to patients who were (very) dissatisfied with their abdominal region but had not undergone abdominoplasty. On the other hand, AE, BAS, and SCW remained the only significantly different body image domains. Again, no differences were found for symptoms of depression and anxiety.

**Table 3:** **Comparison between post-bariatric surgery patients who underwent abdominoplasty and post-bariatric surgery patients who did not undergo abdominoplasty but reported to be very dissatisfied or dissatisfied with their abdominal region**.

	**No abdominoplasty, (very) dissatisfied with abdominal region *N* = 182**	**Abdominoplasty *N* = 54**	**Statistics *t*-tests**
	***M* (*SD*)**	***M* (*SD*)**	
Age	46.66 (11.21)	48.08 (9.69)	ns
BMI current (kg/m^2^)	34.60 (7.23)	32.86 (6.08)	ns
%WL	31.01 (12.13)	37.99 (11.99)	*t* = −3.381, *df* = 233, *p* < 0.001
%EWL	63.72 (29.66)	81.80 (33.89)	*t* = 3.780, *df* = 233, *p* < 0.001
Months since bariatric surgery	40.18 (29.39)	51.31 (28.56)	*t* = 2.405, *df* = 231, *p* = 0.017
Symptoms of anxiety (GAD-7)	6.26 (5.00)	5.29 (4.75)	ns
Symptoms of depression (PHQ-9)	7.82 (5.79)	6.60 (4.80)	ns
IWQOL total^*^	68.57 (27.30)	56.94 (24.10)	*t* = −2.823, *df* = 235, *p* < 0.01
IWQOL physical function^*^	23.93 (11.33)	18.98 (8.52)	*t* = −3.443, *df* = 110.69, *p* = 0.001
IWQOL self-esteem^*^	18.92 (8.29)	15.50 (8.39)	*t* = −2.635, *df* = 234, *p* < 0.01
IWQOL sexual life^*^	9.48 (5.30)	7.77 (4.45)	*t* = −2.355, *df* = 102.23, *df* = 0.02
IWQOL public distress^*^	9.18 (4.90)	7.94 (4.48)	ns
IWQOL work^*^	6.63 (3.42)	5.70 (2.82)	*t* = −2.020, *df* = 103.72, *p* = 0.046
MBSRQ_AE	2.25 (0.71)	2.75 (0.81)	*t* = 4.210, *df* = 229, *p* < 0.001
MBSRQ_AO	3.77 (0.62)	3.58 (0.64)	ns
MBSRQ_FE	3.24 (0.84)	3.39 (0.81)	ns
MBSRQ_FO	3.28 (0.64)	3.26 (0.71)	ns
MBSRQ_HE	3.00 (0.92)	3.16 (0.85)	ns
MBSRQ_HO	3.51 (0.54)	3.49 (0.54)	ns
MBSRQ_IO	2.98 (0.69)	2.79 (0.64)	ns
MBSRQ_BAS	2.66 (0.56)	3.06 (0.60)	*t* = 4.428, *df* = 233, *p* < 0.001
MBSRQ_OP^**^	3.30 (0.75)	3.28 (0.84)	ns
MBSRQ_SCW^**^	4.28 (0.68)	3.90 (0.76)	*t* = −3.454, *df* = 229, *p* = 0.001

* *Lower scores indicate less impairment = better quality of life*.

** *Higher scores indicate more preoccupation with overweight (OP) and a more obese self-perception (SCW); for all other MBSRQ subscales higher scores indicate a more positive body image*.

*Abbreviations: IWQOL, Impact of Weight on Quality of Life-Lite; MBSRQ, Multidimensional Body-Self Relations Questionnaire; AE, Appearance Evaluation; AO, Appearance Orientation; FE, Fitness Evaluation; FO, Fitness Orientation; HE, Health Evaluation; HO, Health Orientation; IO, Illness Orientation; BAS, Body Areas Satisfaction; OP, Overweight Preoccupation; SCW, Self-Classified Weight; %WL, percent weight loss; %EWL, percent excess weight loss*.

Since the post-bariatric surgery patients with and without BCS and specifically with and without abdominoplasty differed with regard to time since bariatric surgery and %EWL we adjusted all analyses for these variables. However, the differences between groups remained significant (results not shown).

### Comparison of MBSRQ subscales between the present BCS sample and healthy control samples from prior studies

**Figure 1:**
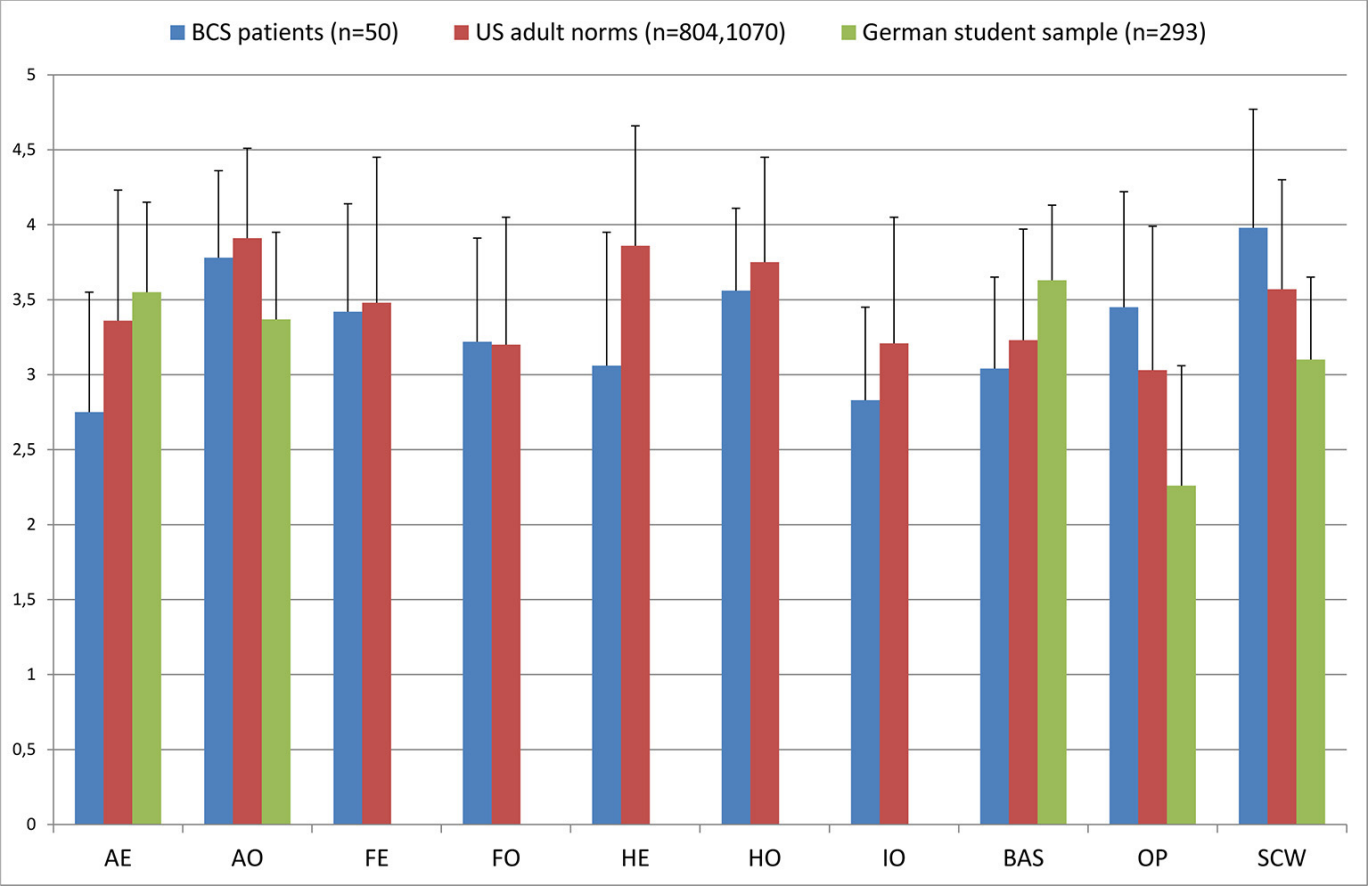
**Comparison of the MBSRQ subscales between female patients after BCS, US female population norms (Cash and Henry, 1995), and healthy German female students (Vossbeck-Elsebusch et al., 2014)**. Abbreviations: AE, Appearance Evaluation; AO, Appearance Orientation; FE, Fitness Evaluation; FO, Fitness Orientation; HE, Health Evaluation; HO, Health Orientation; IO, Illness Orientation; BAS, Body Areas Satisfaction; OP, Overweight Preoccupation; SCW, Self-Classified Weight.

Comparisons with 2 healthy control samples were conducted only for women. The MBSRQ subscales scores of the female BCS subsample were compared with the female adult norms reported by Cash and Henry (1995) and with the scores of the healthy female student group recruited for the German validation of the MBSRQ-AS (Vossbeck-Elsebusch et al., 2014) (Figure 1). Upon visual inspection, the scores of the subscales AE, HE, IO, BAS, OP, and SCW did not reach norm values in our BCS patients. AO, FE, FO, and HO were comparable with the healthy control samples, especially with the adult female norm population.

## Discussion

As expected, post-bariatric surgery patients had significantly less pathological scores on most of the employed scales compared to pre-bariatric surgery patients. Hence, the results of our comparative cross-sectional study support the results of longitudinal studies showing that MWL significantly and positively influences psychosocial aspects including body image (Hrabosky et al., 2006; Karlsson et al., 2007; de Zwaan et al., 2011; Teufel et al., 2012; Burgmer et al., 2014).

Post-bariatric surgery patients who underwent BCS felt more positive and satisfied with their appearance, were more content with the size and appearance of several body areas, and perceived their weight to be less overweight compared to post-bariatric surgery patients who did not undergo BCS. These differences were not a function of %EWL or time elapsed since bariatric surgery. All female patients with breast lifts reported satisfaction with the postoperative results. This finding is in line with results from non-bariatric women receiving breast reductions (Sarwer et al., 2008). Bracaglia et al. (2011) reported that mastopexy after MWL following bariatric surgery produced the best results with regard to body image improvement. Nevertheless, after abdominoplasty and thigh lifts many patients were very dissatisfied or dissatisfied with the final result (27.2 and 40%, respectively). This was also found by others (Bolton et al., 2003; Mitchell et al., 2008; van der Beek et al., 2010). van der Beek et al. (2010, 2012) reported that 33% of patients were very unsatisfied or unsatisfied with the overall result of BCS following bariatric surgery. Importantly, this was not influenced by the occurrence of post-operative complications. Mitchell et al. (2008) reported that some patients found the contoured areas unattractive possibly due to residual deformity or scarring. Comparable to our results Song et al. (2006) reported that improvements in body image satisfaction were regional to the area that underwent treatment. In our study the mid-torso (abdominal) region was the only area on the BAS scale significantly differing between the post-bariatric surgery groups with and without BCS. Finally, Song et al. (2006) suggested that reconstructive surgery may even produce dissatisfaction with the non-contoured parts of the body. Nevertheless, even though not all patients were satisfied with the shape of the contoured body parts, overall, our results and the results of others demonstrate that BCS has a positive impact on appearance related aspects of body image. Thus, dissatisfaction with the contoured body region does not seem to equate with dissatisfaction with the BCS (Sarwer et al., 2008).

The physical complications of excess skin folds result in impairment of physical functioning; improving of physical function is a major aim of BCS. Post-bariatric surgery patients who underwent BCS reported significantly better physical functioning compared to post-bariatric surgery patients who did not undergo BCS. This is in keeping with other studies using other instruments (e.g., SF-36) that demonstrated improvements in physical aspects of quality of life (van der Beek et al., 2010; Azin et al., 2014). In a more detailed analysis Coriddi et al. (2011) found significant improvements in several functional outcomes such as difficulty in personal hygiene, difficulty finding clothes, skin irritation, neck, and abdominal pain in 49 patients who underwent abdominal contouring after MWL.

Interestingly, overall investment in personal appearance (importance of appearance, effort taken to “look good,” and extent of grooming behavior) did not differ between pre- and post-bariatric surgery groups and the scores were similar to population norms. “Looks” were neither more nor less important in either group compared to the population. The effort to “look good,” thus, does not seem to be an important component of patient motivation for body contouring procedures. Similar results were reported by Bolton et al. (2003) who also did not find any changes in overall investment in personal appearance after abdominoplasty in non-bariatric surgery patients. Similarly, illness orientation did not differ between groups and was not different from population norms. Concerns about symptoms of physical illness might be independent of actual body weight and shape, even in severely obese individuals. Compared to pre-bariatric surgery patients the post-bariatric surgery patients reported to feel physically fitter, to value physical fitness more, to feel healthier, and to be more “health conscious.” However, FE and FO as well as HE and HO did not differ between post-bariatric surgery patients with and without BCS indicating that BCS might not have an additional effect on non-appearance related aspects of body image.

There were no differences between post-bariatric surgery participants with and without subsequent BCS in symptoms of depression and anxiety, and in the remaining quality of life and body image domains. The effects of BCS appear to focus primarily on body satisfaction changes and physical functioning but not on general psychosocial functioning (Bolton et al., 2003). Positive post-contouring body image changes are not necessarily matched by similar changes in other aspects of quality of life. E.g., Song et al. (2006) did not find differences in BDI scores before and after BCS in 18 post-bariatric surgery patients. Other studies even found significantly lower (worse) scores on some of the mental aspects of quality of life (SF-36) and no differences between contouring and non-contouring patients on other subscales of the SF-36 (Singh et al., 2012). However, the latter might be explained by a ceiling effect since the differences between pre- and post-bariatric surgery patients were already very large with only little room for further improvement. A wide range of factors contributes to quality of life and symptoms of depression and anxiety with body satisfaction and physical functioning being only two of these many variables. Thus, symptoms of depression and anxiety may have persisted despite positive evaluations of body image changes after BCS.

It is noteworthy, that “matching” patients based on the degree of dissatisfaction/satisfaction with the abdominal region resulted in markedly more differences between post-bariatric surgery patients who did or did not undergo abdominoplasty. This pertains primarily to the quality of life domains Self-Esteem, Sexual Life, and Work Satisfaction. Symptoms of depression and anxiety, and non-appearance related aspects of body image were, again, not different.

Patients after BCS did not reach the values of healthy students and population norms in most of the subscales of the MBSRQ, especially in the subscales pertaining to aspects of appearance. However, it must be kept in mind that the BCS patients in our study were still in the obese range with a mean BMI of 32 kg/m^2^ which is significantly higher than the mean BMI in the healthy student sample and presumably also higher compared to the US adult norm population. Only 24.2% of the BCS patients and 19.5% of the post-bariatric patients without BCS reported a BMI below 28 kg/m^2^. A stable BMI in a relatively low range has been suggested as one prerequisite for body contouring procedures since there is evidence that patients' BMI is a highly significant risk factor for complications (van der Beek et al., 2011; Soldin et al., 2014).

### Strengths

We included a larger sample than reported in most published studies and we included a pre-bariatric surgery comparison group. We investigated a wide range of psychosocial variables using instruments that have been applied before in samples after bariatric surgery and BCS. Even though the MBSRQ has not been specifically developed for bariatric surgery patients, 8 of the 10 MBSRQ subscales differed between pre- and post-bariatric surgery group suggesting that the instrument is sensitive to capture change after bariatric surgery.

### Limitations

This is a cross-sectional study, three different cohorts were compared and we were consequently unable to assess longitudinal changes prospectively in a single sample. It is noteworthy that very few prospective, longitudinal studies have been published so far with mostly rather small sample sizes and high attrition rates (Song et al., 2006; Bracaglia et al., 2011; van der Beek et al., 2012; Modarressi et al., 2013). In addition, except for the pre-bariatric surgery sample this is not a continuous but a self-selected sample, thus, the results may not generalize to other post-bariatric surgery groups.

Time of BCS was not assessed; therefore, we could not investigate whether BCS leads to longer lasting improvements in body image or quality of life. So far, only one study (van der Beek et al., 2012) indicates a sustained quality of life improvement in post-bariatric surgery patients 7 years after BCS. In addition, weight regain after BCS may have compromised the aesthetic result and may have negatively impacted postoperative satisfaction. However, the difference between minimal BMI after bariatric surgery and current BMI was only 2 kg/m^2^ in all patients without differences between patients with and without BCS.

The internal consistency of the OP scale was low with a Cronbach's α of 0.4. Interestingly a similarly unsatisfying result was found in the German validation paper with a Cronbach's α of 0.52 in participants with eating disorders (Vossbeck-Elsebusch et al., 2014). The authors hypothesized that this might be explained by the fact that the scale asks about fat anxiety, weight vigilance, dieting, and eating restraint which might diverge in different weight and shape states. In patients who underwent BCS satisfaction with the contoured regions prior to the operation was assessed retrospectively which has a potential for bias. Finally, the IWQoL-Lite might not be entirely feasible for many post-bariatric surgery patients, since the instrument was developed as an obesity-specific quality of life instrument and most of the questions start with “Because of my overweight…..” However, most patients (93.6%) were still in the overweight and obese range and the IWQoL-Lite has repeatedly been used in post-bariatric surgery patients (Strain et al., 2014). It has been proposed that new psychometrically sound patient-reported outcome instruments are needed for patients undergoing BCS after bariatric surgery that should be amenable to pre- and post-operative administration (Song et al., 2006; Reavey et al., 2011; Klassen et al., 2012; Jabir, 2013).

In summary, the results of our cross-sectional study support the results of longitudinal studies demonstrating significant improvements in different aspects of body image, quality of life, and general psychopathology after bariatric surgery. Also, we found better AE and physical functioning in patients after BCS following bariatric surgery compared to patients with MWL after bariatric surgery who did not undergo BCS. However, we also found a lack of difference in other body image and quality of life domains as well as psychopathological aspects such as symptoms of depression and anxiety. A smaller than expected effect on psychosocial functioning after BCS might be caused by the relatively high complication rate of BCS, the marked scarring, and high expectations which often turn out not to be realistic (van der Beek et al., 2010). A sizable number of our patients after BCS were dissatisfied with the result. Although surgery may improve body contour, it will not result in a perfect body shape. Patients need to be aware that BCS often produces large visible scars, skin irregularities, and residual deformities in body shape. It is of great importance to inform the patients preoperatively and outline realistic expectations. Longitudinal prospective studies with larger sample sizes using valid instruments are warranted. Also interventions modifying expectations might be useful to further improve satisfaction with the results of body contouring procedures.

## Author contributions

All authors contributed to the design, data acquisition and the interpretation of the data. The paper was drafted by Martina de Zwaan, Ekaterini Georgiadou, Martin Teufel, and Astrid Müller and was critically revised by all authors. All authors gave final approval of the version to be published and agreed to be accountable for all aspects of the work in ensuring that questions related to the accuracy or integrity of any part of the work are appropriately investigated and resolved.

### Conflict of interest statement

The authors declare that the research was conducted in the absence of any commercial or financial relationships that could be construed as a potential conflict of interest.
